# Bacterial DNA methylases as novel molecular and synthetic biology tools: recent developments

**DOI:** 10.1007/s00253-025-13442-0

**Published:** 2025-03-06

**Authors:** Carol N. Flores-Fernández, Chris A. O’Callaghan

**Affiliations:** Centre for Human Genetics, Nuffield Department of Medicine, University of Oxford, Roosevelt Drive, Oxford, OX3 7BN UK

**Keywords:** Restriction-modification systems, Targeted methylation, DCas9/gRNA, DNA assembly, Bacterial transformation, Crop engineering

## Abstract

**Abstract:**

Bacterial DNA methylases are a diverse group of enzymes which have been pivotal in the development of technologies with applications including genetic engineering, bacteriology, biotechnology and agriculture. This review describes bacterial DNA methylase types, the main technologies for targeted methylation or demethylation and the recent roles of these enzymes in molecular and synthetic biology. Bacterial methylases can be exocyclic or endocyclic and can exist as orphan enzymes or as a part of the restriction-modifications (R-M) systems. As a group, they display a rich diversity of sequence-specificity. Additional technologies for targeting methylation involve using fusion proteins combining a methylase and a DNA-binding protein (DNBP) such as a zinc-finger (ZF), transcription activator-like effector (TALE) or CRISPR/dCas9. Bacterial methylases have contributed significantly to the creation of novel DNA assembly techniques, to the improvement of bacterial transformation and to crop plant engineering. Future studies to define the characteristics of more bacterial methylases have potential to identify new tools of value in synthetic and molecular biology and with widespread applications.

**Key points:**

*• Bacterial methylases can be used to direct methylation to specific sequences in target DNA*

*• DNA methylation using bacterial methylases has been applied to improve DNA assembly and to increase the efficiency of bacterial transformation*

*• Site-selective methylation using bacterial methylases can alter plant gene expression and phenotype*

## Introduction

**Fig. 1:**
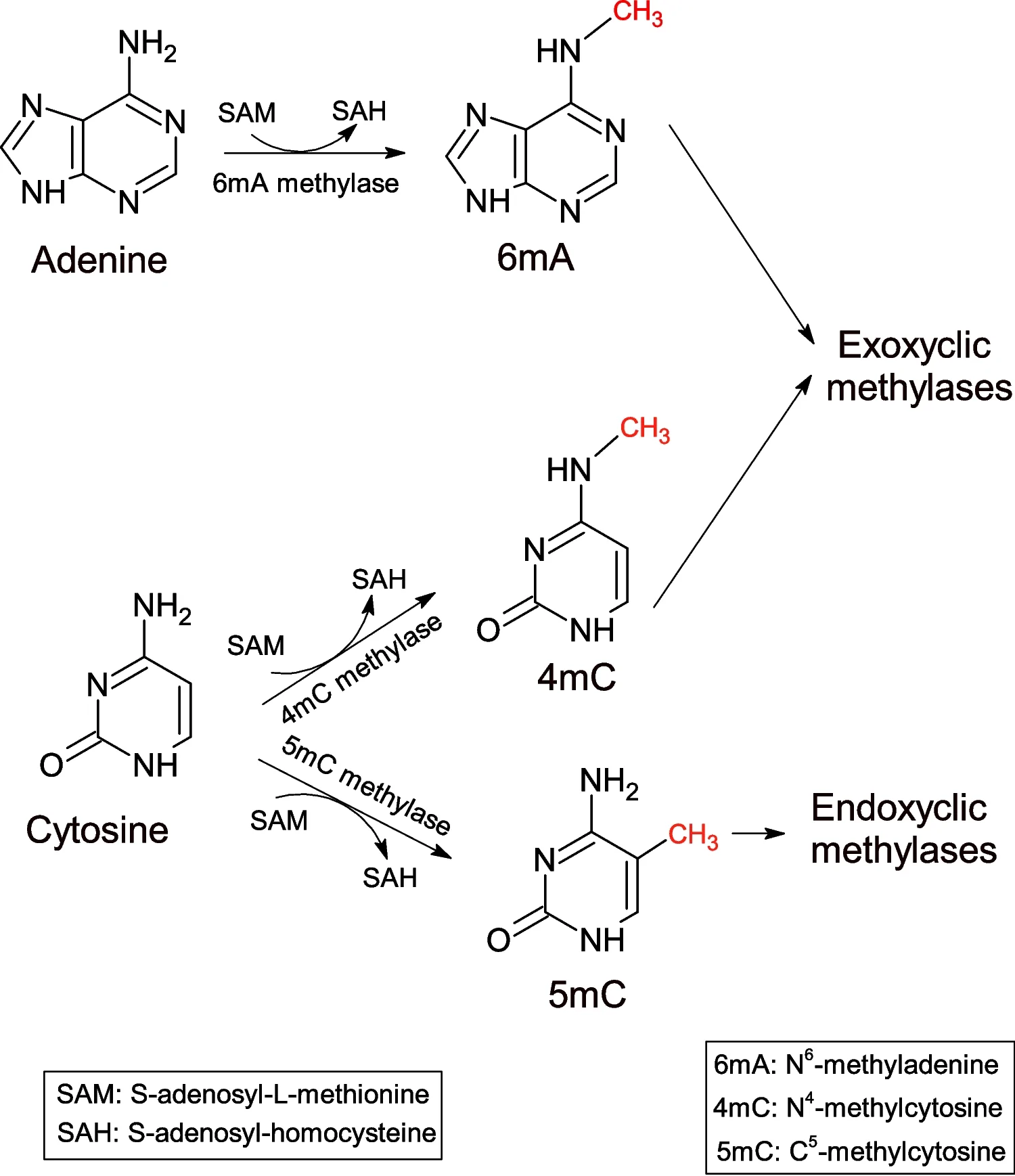
Bacterial DNA methylases classified according to their mode of action

Bacterial DNA methylases have acquired a fundamental role in the development of a number of molecular and synthetic biology tools. These enzymes use S-adenosyl-L-methionine (SAM) as a methyl group donor for DNA methylation (Papaleo et al. 2022; Abdelraheem et al. 2022). Bacterial DNA methylases can be classified as exocyclic or endocyclic according to their mode of methylation (Fig. 1). The exocyclic methylases transfer the methyl group from SAM to an exocyclic amino group outside the ring structure at position 4 in cytosine: N^4^-methylcytosine (4mC) methylases or position 6 in adenine: N^6^-methyladenine (6 mA) methylases. The endocyclic methylases transfer the methyl group from SAM to a carbon within the ring structure at position 5 in cytosine: C^5^-methylcytosine (5mC) methylases (Beaulaurier et al. 2019; Ren et al. 2022; Gao et al. 2023). Deamination of C^5^-methylcytosine produces thymine which causes frequent mutations despite repair systems (Casadesús and Sánchez-Romero 2022). Bacterial DNA methylases have been identified as orphan solitary enzymes or as part of the restriction-modification (R-M) systems. Orphan methylases are generally conserved and can regulate DNA replication and repair as well as gene expression. The most widely studied have been Dam, CcrM and Dcm (Horton et al. 2019; Mehershahi and Chen 2021; Papaleo et al. 2022; Gao et al. 2023) (Table 1). R-M systems can regulate gene expression and play an important function in bacterial immunity by protecting host DNA from cleavage by restriction enzymes deployed to cleave foreign DNA. Methylases in the R-M systems are associated with a corresponding restriction enzyme; thus, they act by methylating the host bacterial DNA while the restriction enzyme digests the foreign unmethylated DNA (Mattei et al. 2022; Papaleo et al. 2022; Ren et al. 2022).

**Table 1:** Most widely studied orphan solitary DNA methylases

Enzyme	Source	Functional protein	Methylation position	Recognition sequence and methylated base*	Preferred target DNA
Dam	*Escherichia coli*	Monomer	N^6^-adenine	5′-G**A**^m6^TC-3′ 3’-CT**A**^m6^G-5’	Unmethylated or hemimethylated
CcrM	*Caulobacter crescentus*	Dimer	N^6^-adenine	5′-G**A**^m6^NTC-3′ 3’-CTN**A**^m6^G-3’	Hemimethylated
Dcm	*Escherichia coli*	Dimer	C^5^-cytosine	5′-C**C**^m5^AGG-3′ 3’-GGT**C**^m5^C-5’ 5′-C**C**^m5^TGG-3′ 3’-GGA**C**^m5^C-5’	Unmethylated or hemimethylated

^*^Methylated base in bold

**Fig. 2:**
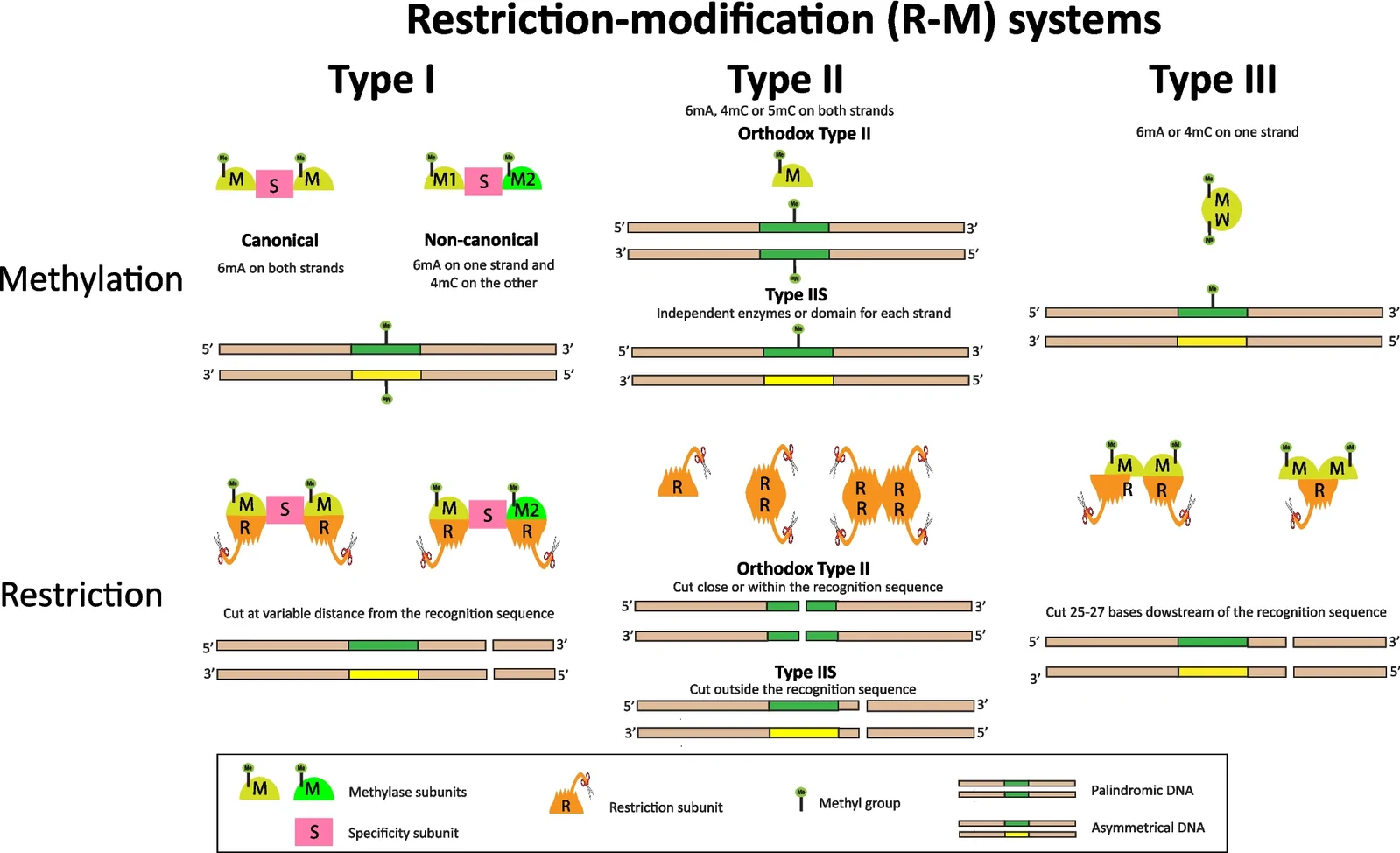
Restriction-modification (R-M) systems: methylation and restriction features

Three types of R-M systems involving methylases have been described whose classification is based on the enzyme structure, subunit composition and target DNA for methylation and restriction (Table 2 and Fig. 2). Type I and type III systems are formed by single enzymes with the subunits M (methylation), R (restriction) and S (specificity) for type I; and M and R for type III (Beaulaurier et al. 2019; Gao et al. 2023). Type I methylases normally use two identical M subunits (M_2_) for 6 mA modification in both DNA strands. However, non-canonical type I methylases have been discovered which use two different M subunits (M1M2) for 6 mA modification on one strand and 4mC on the other (Zhu et al. 2022). In type I R-M systems, methylases introduce methyl groups on both strands of an asymmetric DNA sequence preferring hemimethylated substrates, and restriction enzymes cut the DNA at variable distances from their recognition sequence (Tock and Dryden 2005; Gao et al. 2023). In type III R-M systems, methylases generally catalyse 6 mA reactions, but a recently reported subgroup catalyses 4mC modifications (Murray et al. 2021). Type III methylases introduce methyl groups on only one strand of asymmetric DNA and the restriction enzymes cut 25–27 bases downstream of their recognition sequence. Most type II R-M systems involve two separate enzymes, one methylase (M) and one restriction enzyme (R), which act independently but usually recognize the same sequence. Type II methylases can catalyse 6 mA, 4mC and 5mC reactions and orthodox enzymes act on both strands in palindromic DNA. Type II restriction enzymes cut close to (Type IIS) or within their recognition sequence (Type IIP) (Murray 2000; Roberts et al. 2003; Tock and Dryden 2005; Beaulaurier et al. 2019; Mehershahi and Chen 2021; Gao et al. 2023). Additional classes of the type II R-M systems have been described including of type IIS. This class can include two independent methylases or chimeric methylases composed of two fused independent domains each acting on one strand in asymmetric DNA and exhibiting variable preferences for unmethylated or hemimethylated substrates (Roberts et al. 2003; Furmanek-Blaszk et al. 2009; Madhusoodanan and Rao 2010; Fokina et al. 2023; Kennedy et al. 2023) (Fig. 2). Enzymes belonging to the type II R-M systems have been the most studied and commercialized, with applications in genetic engineering, biotechnology and molecular and synthetic biology (Murray et al. 2021; Fokina et al. 2023; Gao et al. 2023). The REBASE database (https://rebase.neb.com/rebase/rebase.html) includes around 11,000 methylases and 5000 restriction enzymes corresponding to the three types of R-M systems. Of these enzymes, approximately 3000 of each belong to the type II R-M systems (Roberts et al. 2023).

**Table 2:** Types of Restriction-Methylation (R-M) systems including methylases and their characteristics

R-M system	Structure	Subunits	Methylation functional protein	Methylation position	Restriction functional protein	Restriction site	Target DNA
Type I	One enzyme	M, R and S	M_2_S M1M2S	N^6^-adenine N^4^-cytosine	R_2_M_2_S R_2_M1M2S	Variable positions from the recognition sequence	Long bipartite asymmetrical
Type II	Two individual enzymes	M or R	M	N^6^-adenine N^4^-cytosine C^5^-cytosine	R, R_2_ or R_4_	Close or within the recognition sequence	Short palindromic Short asymmetrical
Type III	One enzyme	M (Mod) and R (Res)	M_2_	N^6^-adenine N^4^-cytosine	R_2_M_2_ R_1_M_2_	25–27 downstream bases from the recognition sequence	Short asymmetrical

*M*, methylation; *R*, restriction; *S*, specificity. M_2_, R_2_ and R_4_ denote multiple identical subunits; M_1_ and M_2_ denote different subunits. Bipartite, divided into two DNA molecules

Recent reviews have considered the classification, structure and use of bacterial DNA methylases (Ren et al. 2022; Gao et al. 2023; Chang et al. 2024; Seem et al. 2024; Wong and Yim 2024). Here we review the key technologies for targeting DNA methylation, focusing on the role of bacterial methylases in the development of molecular and synthetic biology tools. This includes the applications of bacterial methylases in DNA assembly, in improving bacterial transformation efficiency and in plant gene expression regulation and engineering.

## Technologies for targeted DNA methylation

Methylation or demethylation can be targeted to specific DNA sequences using programmable DNA-binding proteins (DNBPs) such as zinc-finger (ZF), transcription activator-like effector (TALE) or CRISPR/Cas9 proteins fused to a methylase or demethylase enzyme (Zhu et al. 2024).

ZF proteins are structurally and functionally diverse eukaryotic transcription factors which coordinate one or more zinc ions. Synthetic ZF modules of 30 amino acids that interact with three consecutive bases in the major groove can be engineered and linked in tandem (finger domains) to bind with high specificity to target DNA rich in GNN sequences. These engineered ZF domains can typically bind 18 or more bases in the target DNA and libraries of ZF modules have been created to bind each of the 64 possible base triplets (Thakore and Gersbach 2015; Negi et al. 2023; Kamaliyan and Clarke 2024).

TALEs are prokaryotic proteins which can bind specific DNA sequences through central repeat regions of 34 amino acids arranged in tandem. The base specificity of each repeat is defined by the amino acids at positions 12 and 13, which are known as the repeat-variable di-residues (RVD). The RVDs NG, NI, NN, HD, and NS recognize the bases T, A, G/A, C, and A/G/C/T respectively, of which HD and NN are recognized as ‘strong’ and appear to convey higher affinity DNA-binding. Each repeat binds only one nucleotide, so multiple repeats must be assembled according to the length of the target nucleotides (usually 12–18) in a target sequence. Tools and kits for designing synthetic TALEs have been developed in order to improve this technology (Streubel et al. 2012; Schulze and Lammers 2021; Becker and Boch 2021; Shamshirgaran et al. 2022).

CRISPR/Cas9 tools are built using the *Streptococcus pyogenes* Cas9 nuclease which can be targeted to bind to a specific DNA sequence. This specific binding requires a guide RNA (gRNA) which consists of a crRNA, tracrRNA and a variable 20-nucleotide target-specific sequence complementary to the desired target sequence. The gRNA binds to Cas9, guiding it to the target DNA. Target DNA binding also requires a protospacer-adjacent motif (PAM) on the strand to which the gRNA is complementary. The PAM sequence for the *S. pyogenes* CRISPR/Cas9 system is NGG (Zhang et al. 2019; Bhardwaj and Nain 2021). CRISPR/Cas9 systems are generally simpler, faster and more cost-effective to develop than ZFs or TALEs (Thakore and Gersbach 2015; Pflueger et al. 2019).

**Fig. 3:**
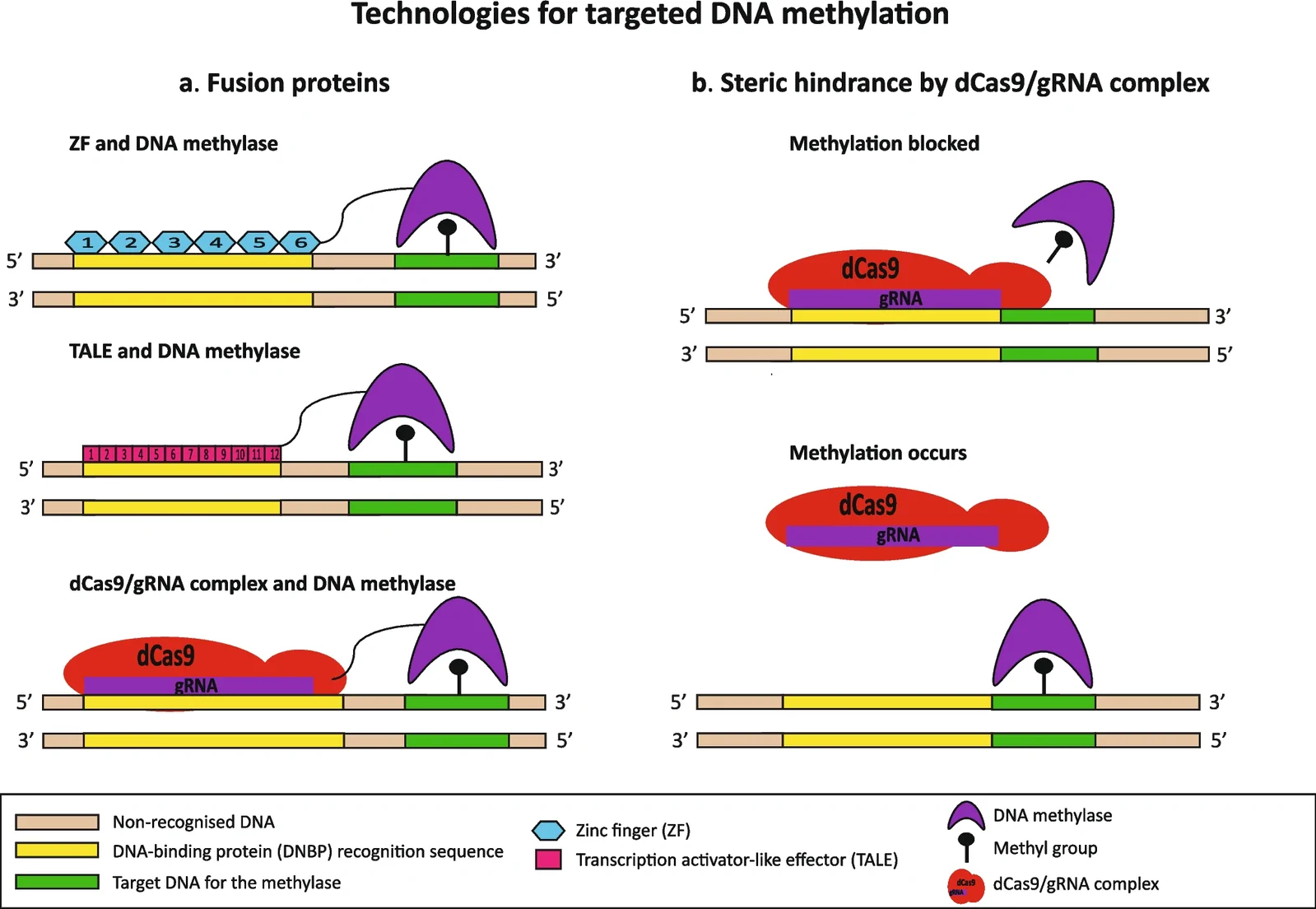
Technologies for targeted DNA methylation. **a** Fusion proteins formed by DNA-binding proteins (DNBP) such as zinc finger (ZF), transcription activator-like effector (TALE) and CRISPR/Cas9 (dCas9) combined with a methylase. **b** Steric hindrance by dCas9/gRNA complex. Methylation by the methylase is blocked when the dCas9/gRNA complex is bound to the target sequence, but methylation can occur when it is not bound. A demethylase could be blocked in a similar manner

These DNBPs (ZF, TALE and CRISPR/Cas9) can each be custom-designed to target for methylation or demethylation to a specific DNA sequence location. The DNBP can be engineered as a fusion protein fused to a methylase or demethylase enzyme. The DNBP component binds to specific bases within the target DNA sequence and so brings the fused enzyme to this location. Fusion proteins for diverse applications have been constructed by combining DNBPs with methylases, or ten-eleven translocation (TET) enzymes implicated in DNA demethylation (Pflueger et al. 2019; Yano and Fedulov 2023; Zhu et al. 2024) (Fig. 3a).

The Cas9 system has been further modified by the introduction of two mutations which inactivate its nuclease activity, allowing it to bind to DNA sequences defined by the gRNA without cleaving the DNA. This deactivated Cas9 is referred to as dCas9. Targeted blockade of methylation has been achieved simply by the steric hindrance arising when a dCas9/gRNA complex binds to a target DNA sequence. Using this approach, a methylase enzyme can methylate other DNA sequences, but the sequence to which the gRNA is complementary is bound by dCas9 which blocks the enzyme’s access to this sequence and so protects the sequence from methylation (Sapozhnikov and Szyf 2021, 2022, 2023) (Fig. 3b). Limitations of DNA-binding technologies include off-target activity, competition and interactions with endogenous proteins, and delivery complications when in cells (Sapozhnikov and Szyf 2023; Zhu et al. 2024) which can be overcome when appropriate by performing the reactions using recombinant enzymes (Flores-Fernández et al. 2024).

## Development of DNA assembly techniques using methylases

DNA assembly techniques are important tools in molecular biology, synthetic biology and biotechnology. The key developmental aim in this research is an approach to build long DNA constructs, ideally in a one-pot reaction without any sequence constraint and without the introduction of unwanted scar sequences at junctions between assembled DNA molecules. Several assembly techniques have been developed based on the Golden Gate or Gibson methods and methylases have been applied with success to improve the Golden Gate method (Lin and O’Callaghan 2018, 2020). Golden Gate-based methods use type IIS restriction enzymes to release DNA fragments to be assembled from donor plasmids and to digest the assembly vectors (acceptor plasmids). Since type IIS endonucleases cut outside their recognition sequence, the generated overhangs are variable in nucleotide composition, so diverse fragments with compatible overhangs can be assembled without reconstituting the original type IIS restriction site (Bird et al. 2022; Sikkema et al. 2023). Thus, methods such as Modular Cloning (MoClo) (Weber et al. 2011; Marillonnet and Werner 2019; Marillonnet and Grützner 2020), PS-Brick (Liu et al. 2019), Star-Stop assembly (Taylor et al. 2019) and Golden Gate with Data-optimized Assembly Design (GG-DAD) (Pryor et al. 2020, 2022; Sikkema et al. 2023) have been described. These methods have demonstrated important achievements within the assembly tools. Constructions of up to 33 kb have been assembled through the modular assembly of the fragments (Weber et al. 2011). Also, assemblies of up to 40 kb have been constructed by using many short linear fragments amplified by PCR and assembled in one round one-pot reactions (Pryor et al. 2022). Likewise, type IIP and IIS endonucleases have been used in combination for the assembly of metabolic pathway genes (Liu et al. 2019). Finally, conserved sequences such as start and stop codons for CDSs have been used as fusion sites for the functional assembly of expression units (Taylor et al. 2019). Despite these achievements, drawbacks remain to be addressed including the need for multiple vectors and fusion sites and the need for more than one type IIS restriction enzyme for sequential rounds of hierarchical assemblies, but the key problems are the unwanted sequence scars resulting from hierarchical assembly and the incapacity of these approaches to assemble sequences that contain internal sites for the type IIS restriction enzymes used in the assembly (Weber et al. 2011; Liu et al. 2019; Taylor et al. 2019; Pryor et al. 2022).

**Fig. 4:**
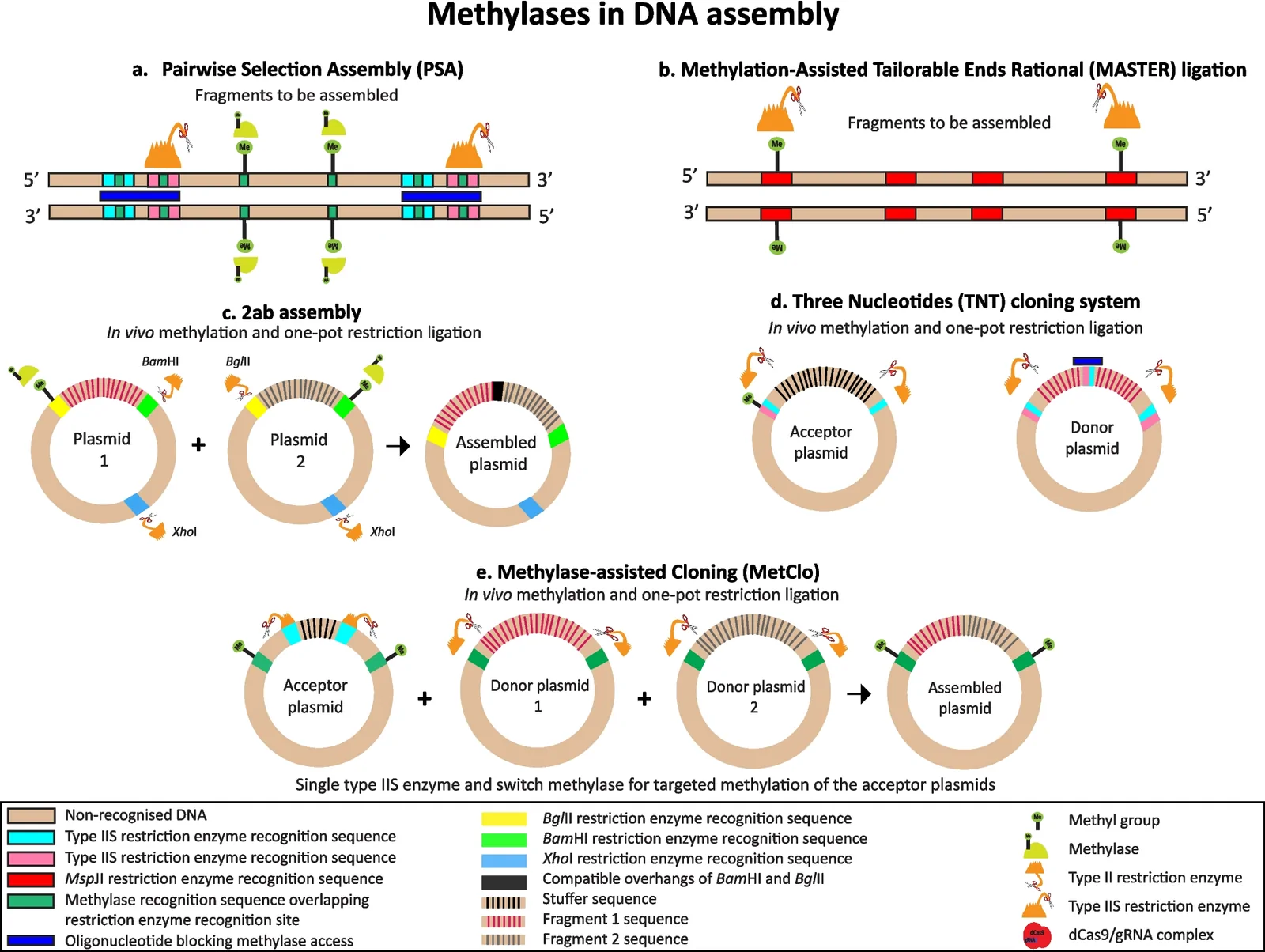
Uses of methylases in DNA assembly. **a** Pairwise Selection Assembly (PSA), green bars represent potential sites of methylation and these are blocked in the flanking restriction sites (pink and light blue) by the oligonucleotide polymers (dark blue). **b** Methylation-Assisted Tailorable Ends Rational (MASTER) ligation, the *Msp*JI restriction enzyme only cuts methylated recognition sites so leaves unmethylated internal sites uncut. **c** 2ab assembly, different site-specific methylases are used to inactivate a *Bgl*II site in plasmid 1 and a *Bam*HI site in plasmid 2 respectively. *Bgl*II and *Bam*HI produce compatible overhangs. **d** Three Nucleotides (TNT) cloning system, a methylase is used to inactivate type IIS restriction enzyme sites. **e** Methylase-assisted Cloning (MetClo), a switch methylase is used to methylate and block the pair of outer type IIS restriction enzyme sites in the acceptor plasmid. This outer restriction enzyme site is engineered to partially overlap with a recognition site for the methylase which does not recognise the restriction enzyme site alone

Bacterial methylases and DNA methylation have been used in efforts to overcome these drawbacks. Assembly methods using methylases include Pairwise Selection Assembly (PSA) (Blake et al. 2010), Methylation-Assisted Tailorable Ends Rational (MASTER) ligation (Chen et al. 2013), 2ab assembly (Leguia et al. 2013), Three Nucleotides (TNT) cloning system (De Paoli et al. 2016), Methylase-assisted Cloning (MetClo) (Lin and O’Callaghan 2018) and the application of methylation to a standardized Golden Gate-type BioBrick assembly (Matsumura 2020, 2022) (Fig. 4).

In PSA, *M.Sss*I methylates the C^m5^G sequence in any internal type IIS restriction sites in the fragments to be assembled. Prior to this, the flanking sites required for the assembly are blocked from methylation by RecA-oligonucleotide polymers. This method allows the sequential assembly of pairs of DNA fragments and has been used to assemble a 91 kb fragment containing 19 internal type IIS restriction sites without the need to introduce mutations in the sequences (Blake et al. 2010) (Fig. 4a).

In MASTER ligation, the modification-dependent endonuclease *Msp*JI that recognizes the methylated sequence C^m5^NNR and cuts N9/N13 from the 3’ of the methylated cytosine is used to generate 4 bp overhangs during DNA assembly. PCR with methylated primers is used to introduce methylation into the flanking sites of fragments to be assembled. During the assembly reaction, *Msp*JI cuts the methylated flanking sites only, leaving any unmethylated *Msp*JI recognition sequences within the sequence undigested. Thus, this method allows the assembly of fragments containing internal sites for the *Msp*JI restriction enzyme used in the assembly and has been used to assemble a construct of around 29 kb from *Streptomyces coelicolor* (Chen et al. 2013) (Fig. 4b).

In 2ab assembly, two fragments are assembled in the donor plasmids, each fragment is flanked at its 5’ end by a *Bgl*II site and at its 3’ end by a *Bam*HI site; both plasmids also contain an *Xho*I site. The donor plasmid containing the 5’ (left) fragment is propagated in an *Escherichia coli* strain expressing a methylase that methylates and inactivates only the *Bgl*II site whereas the donor plasmid containing the 3’ (right) fragment is propagated in an *E. coli* strain that expresses a methylase that methylates and inactivates only the *Bam*HI site. When the two plasmids are combined in one pot and exposed to *Bgl*II, *Bam*HI and *Xho*I, the 3’ end of the left fragment is cut by *Bam*HI and the 5’ end of the right fragment is cut by *Bam*HI. As *Bgl*II and *Bam*HI cleavage results in compatible cohesive overhangs, these junctions can be ligated to join the two fragments together and when the *Xho*I sites from each plasmid are also ligated, the result is a new plasmid containing the combined fragment. A total of 528 plasmids with a success rate of 96% were constructed using this method (Leguia et al. 2013). In vivo site-specific methylation of the donor and acceptor plasmids used for the assembly was applied in a Golden Gate-type Biobrick assembly (Matsumura 2022) (Fig. 4c).

In TNT cloning, donor and acceptor plasmids contain sites for the type IIS restriction enzymes *Ear*I and *Lgu*I which are used in the assembly. The recognition site for *Ear*I is included within the recognition site for *Lgu*I. As part of the protocol, *E. coli* expressing the *M.Taq*I methylase was used to methylate and inactivate *Ear*I sites within plasmids, thus preventing subsequent digestion of these plasmids by *Ear*I. In addition, internal *Lgu*I and *Ear*I sites in the fragments to be assembled were blocked from digestion by these enzymes using oligonucleotides (De Paoli et al. 2016) (Fig. 4d).

In MetClo, methylation is used to adapt MoClo so that only one type IIS restriction enzyme is required instead of two. Acceptor plasmids have two outer and two inner type IIS restriction sites as implemented in MoClo, but all these sites are for one enzyme. The outer flanking sites are designed, so that they are recognized and methylated by ‘switch methylases’ and so inactivated or ‘switched’ off. During an assembly reaction, one type IIS restriction enzyme is used to cut the unmethylated inner sites of the acceptor plasmid and to release the fragments to be assembled from the donor plasmids. The assembled plasmid with the methylated outer sites can then be propagated in a methylase-free bacterial strain to remove methylation and used as a new donor plasmid in the next round of a hierarchical assembly. Long constructs up to 218 kb have been assembled using MetClo (Lin and O’Callaghan 2018) (Fig. 4e). MetClo used methylases from the type II R-M systems associated with their corresponding type IIS restriction enzymes. Our group has continued the study of these methylases to expand their applications for the development of assembly techniques and to use these methylases in recombinant form in vitro. Recognition sequences of switch methylases partially overlap with those of the corresponding restriction endonucleases permitting the engineering of these restriction enzyme recognition site sequences for site-selective methylation and restriction. Besides switch methylases, we studied what we termed ‘non-switchable methylases’ and have cloned and expressed recombinant switch and non-switchable methylases associated with the type IIS endonucleases *Bsa*I, *Bpi*I and *Lgu*I for use in vitro. Recognition sequences of switch methylases partially overlap with those of the endonucleases permitting the engineering of these sequences for site-selective methylation and restriction. Recognition sequences of non-switchable methylases fully overlap with those of their associated restriction endonuclease allowing the methylation of all the endonuclease recognition sequences (Flores-Fernández et al. 2024). In this way, non-switchable methylases could be used for targeted methylation of the internal type IIS restriction sites of the fragments to be assembled while the flanking sites could be selectively protected from methylation by the steric hindrance from a DNBP.

Overall, all these assembly techniques demonstrate that methylases and DNA methylation are becoming pivotal for developing novel, efficient and universal DNA assembly tools.

## Improving bacterial transformation using methylases

**Fig. 5:**
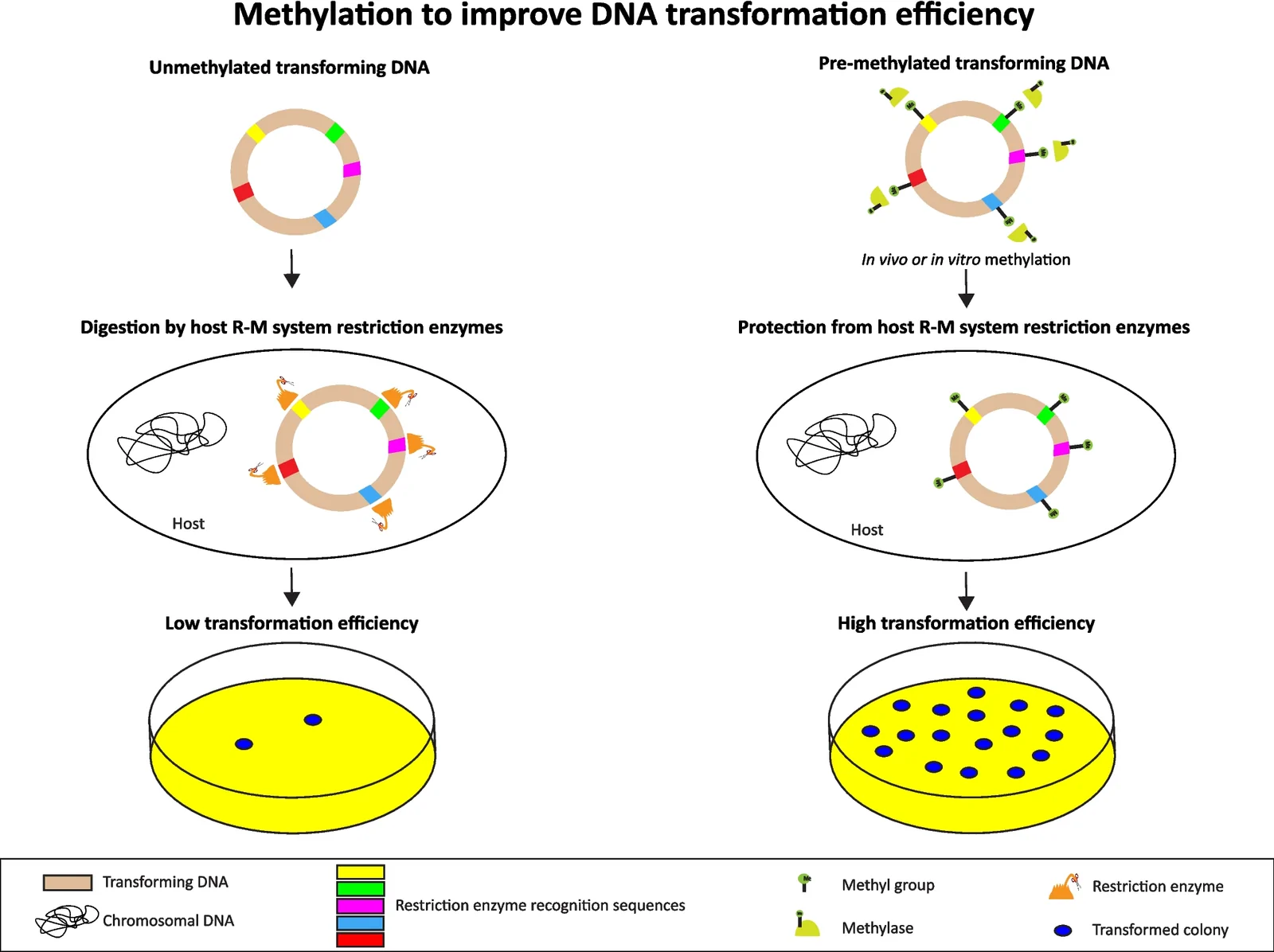
Effect of pre-methylation of transforming DNA to increase transformation efficiency

Methylases have been used to improve bacterial transformation efficiency by preventing digestion of the exogenous DNA by host R-M system restriction enzymes and so improving DNA stability (Won and Yim 2024). During bacterial transformation, exogenous DNA is taken into the cell and then either incorporated into the bacterial genome or replicated independently (Ren et al. 2019). As exogenous DNA is highly vulnerable to digestion by host R-M system restriction enzymes, methylases have been used to inhibit this digestion by methylation of the exogenous DNA before the transformation (Hu et al. 2023; Won and Yim 2024) (Fig. 5). This pre-methylation step can improve transformation efficiency. Key considerations in planning this pre-methylation include the methylation pattern of the exogenous DNA, the methylome pattern of the host DNA, the selection of the appropriate methylase(s) and the type of methylation reaction to be used. The chosen methylase must introduce a methyl group within the recognition sequences of the host R-M system endonucleases to inhibit digestion of the transforming DNA by these endonucleases. This inhibition will occur when the methylase recognition sequence overlaps the endonuclease recognition sequence. If the sites do not overlap, the restriction enzyme recognition sequence will not be methylated and will be vulnerable to digestion which will reduce transformation efficiency. In vitro methylation reactions using recombinant enzymes are generally preferred as they have been shown to be more efficient and straightforward than in vivo methylation (Guss et al. 2012; Ren et al. 2022).

The effect of DNA methylation on the transformation of bacterial hosts has been studied in both clinical and industrial contexts, especially in situations where genetic manipulation is challenging. *Campylobacter jejuni* has a transformation system methyltransferase (CtsM) consisting of an orphan methylase that introduces methyl groups in the conserved motif RA^m6^ATTY on self and foreign DNA. In vitro methylation of exogenous DNA by this enzyme strongly increases the efficiency of transformation of the DNA into *C. jejuni* (Beauchamp et al. 2017). *Acinetobacter baumannii* strain A118 has a specific methylase that introduces methyl groups in the motif RGATCY on self-DNA only, protecting it from endonuclease digestion. This methylation allows the selective transformation of self-DNA while unmethylated DNA from other related strains is degraded by the host endonucleases (Vesel et al. 2023). The *E. coli* Dam methylase methylates adenine in the sequence motif GATC and DNA that has been methylated in vitro by Dam methylase is also efficiently transformed by *A. baumannii* strain A118, presumably because GATC is part of the RGATCY motif (Vesel et al. 2023; Soler-Bistué 2023). *Haemophilus parasuis* is a physiologically and genetically diverse bacterium with strain-specific R-M systems. The transformation efficiency of shuttle plasmids varies nearly 40-fold when the plasmids are methylated using cell-free extracts from different *H. parasuis* strains, with the greatest efficiency occurring when the lysate matches the strain being transformed (Zhang et al. 2018). Similarly, *Helicobacter pylori* carries multiple types of R-M systems that make genetic manipulation challenging, but pre-methylation of plasmids in vitro using *H. pylori* lysates from different strains enhances bacterial transformation efficiency, with the greatest efficiency occurring when the lysate matches the strain being transformed (Zhao et al. 2018). In *S. pyogenes*, the transformation efficiency of plasmids that were pre-methylated in vivo was increased by genetic deletion of the host R subunit alone or of the host R, M and S subunits together. The transformation efficiency was higher when only the R subunit was deleted compared to the deletion of the R, M and S subunits together, suggesting that the active M and S subunits enhanced transformation efficiency by methylating additional sites on the plasmid (Nye et al. 2019). Tick-borne *Borrelia* species can result in Lyme disease and have effective R-M systems. The efficiency of the transformation of plasmids into *Borrelia burgdorferi* has been shown to be enhanced by pre-methylation of the plasmids in vitro using methylases from the *Borrelia* type II R-M systems (Ruivo et al. 2024).

Lactic acid bacteria (LAB) have a wide range of applications in industry, but genetic modification is limited by their poor capacity for stable uptake of exogenous DNA. The effect of methylation on transformation has been tested by preparing shuttle plasmids in *E. coli* strains expressing different Dam and Dcm profiles. The effects of methylation on the transformation efficiency varied, with Dam methylation altering the efficiency of transformation into some, but not all LAB strains tested. In contrast, Dcm methylation did not affect transformation efficiency in the strains tested (Welker et al. 2020). Species of the genus *Methylomonas* have been identified as potential candidates for the production of biofuels and complex molecules such as carotenoids, but their R-M systems complicate genetic engineering. In this context, the methylase AYM39_01025 was identified in the *Methylomonas* sp. strain DH-1 and shown to methylate the sequence TGGC^m5^CA. *E. coli* transformed with this methylase was used to pre-methylate a plasmid harbouring a gene involved in carotenoid production. Transformation of this methylated plasmid into *Methylomonas* was 124% more efficient than transformation with the unmethylated plasmid and resulted in enhanced carotenoid production (Ren et al. 2020).

The Imitating Methylation Patterns Rapidly in Transcription-Translation (IMPRINT) approach has been developed to enhance transformation in gram-positive and gram-negative bacteria. With this approach, methylases from the R-M systems of host bacterial species are cloned and expressed in a cell-free transcription-translation reaction. Plasmids to be pre-methylated are incubated in a one-pot reaction with the product of these transcription-translation reactions which contains the methylases to recreate the host methylation pattern in the plasmids. These pre-methylated plasmids evade degradation by the corresponding restriction enzymes and this enhances the efficiency of transformation. As R-M systems can be strain-specific, the efficiency depends on the extent of the match between the methylases used in the transcription-translation reaction and the methylases in the strain to be transformed. A high-throughput version of the IMPRINT approach has been used with bar-coded plasmids and next-generation sequencing to rapidly test which combinations of methylases provide the greatest transformation efficiency for any given strain (Vento et al. 2024).

## Plant genetic and crop engineering research using methylases

DNA methylation is an important epigenetic modification in plants and has the potential to influence genome stability, the expression of genes and transposable elements, plant growth and development and resistance to environmental stress (Kumar and Mohapatra 2021; He et al. 2022; Talarico et al. 2024). Plant methylation is controlled by specific pathways that perform de novo and maintenance methylation. Methyl groups can form 5mC or 6 mA in CG, CHG and CHH (H = A, T or C) sequence motifs which can be symmetrical or asymmetrical (Leichter et al. 2022). The most common form of methylation is 5mC, which has been widely studied and can occur in all three sequence elements (Liu et al. 2023). A number of tools for targeted DNA methylation in plants have been developed for agricultural purposes. The main tools include fusion proteins that combine an enzyme that affects methylation with a DNBP such as a ZF, TALE or CRISPR/Cas9 complex. Plant and other eukaryotic enzymes that alter methylation have typically been used in these tools, but bacterial methylases have also shown valuable potential for the genetic engineering of plants. The effects of methylation depend on the methylation site; thus, promoter methylation is associated with gene silencing and repression, transcription start site methylation with transcription initiation delays and gene body methylation can have variable effects (Chang et al. 2024; Seem et al. 2024). The main organism studied in this context is *Arabidopsis thaliana,* but recent studies have included other non-model plants (Chen et al. 2022).

**Fig. 6:**
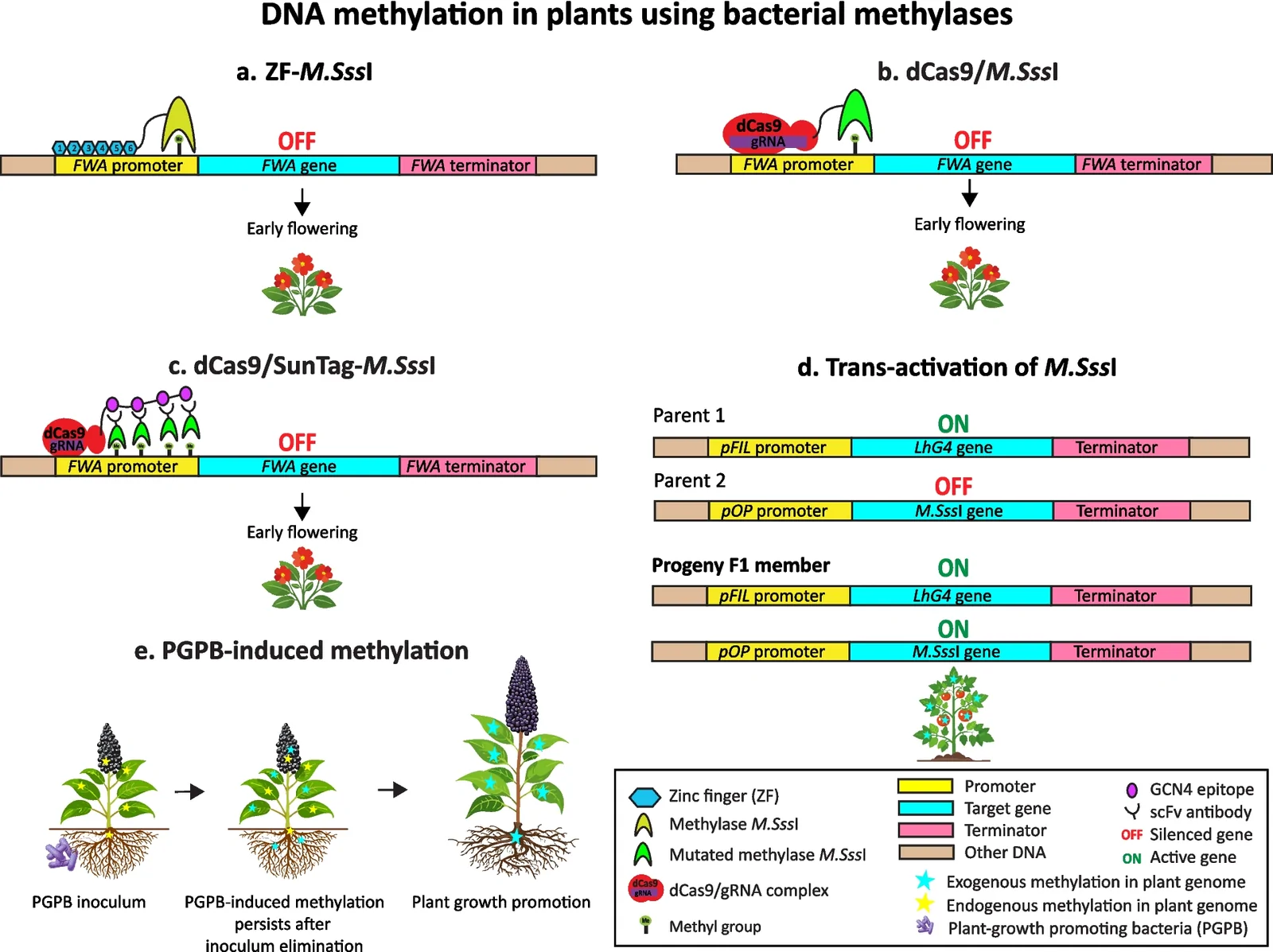
DNA methylation tools in plants using bacterial methylases and their effects. **a** Methylation using the methylase *M.Sss*I can be targeted using zinc finger. **b** A mutated form of *M.Sss*I with reduced off-target activity and targeted by dCas9/gRNA. **c** Recruitment of multiple methylase molecules to the target site using a SunTag. **d** Heritable transactivation of *M.Sss*I methylation. **e** Persistent methylation induced by plant-grown promoting bacteria

Fusion proteins formed by DNBPs and bacterial CG-specific methylases have been designed for targeted DNA methylation in *A. thaliana*. The methylase *M.Sss*I from the bacterium *Mollicutes spiroplasma* (strain MQ1) is a methylase of 386 amino acids which methylates the dinucleotide C^m5^G. A synthetic ZF protein was fused with *M.Sss*I to target the promoter of the *FWA* gene, which is associated with late flowering. ZF-*M.Sss*I methylated the *FWA* promoter causing early flowering as a result of gene silencing (Fig. 6a). The methylation was inherited by subsequent generations in the absence of *M.SssI* through activity of the endogenous pathways that maintain methylation. However, multiple off-target sites throughout the genome were found to be heritably methylated due to non-specific binding and methylation by ZF-*M.SssI* (Liu et al. 2021). CRISPR/dCas9 technology was applied to this approach to minimize the number of off-target sites, and a fusion protein was created in which position 147 of *M.Sss*I was mutated from glutamine to leucine to decrease its methylase activity and reduce non-specific methylation (Fig. 6b). This dCas9/*M.Sss*I fusion protein efficiently methylated the *FWA* promoter with minimum off-target sites. A dCas9/SunTag system has also been developed to enhance methylation efficiency (Ghoshal et al. 2021). The dCas9 is fused to a SunTag, a polypeptide tail consisting of a repeating peptide epitope separated by linker sequences. The methylase is fused to a single-chain fragment (scFv) antibody that binds with specificity to the peptide epitope. The antibody-epitope interaction allows the dCas9/SunTag system to recruit multiple methylase molecules to the target DNA site (Tanenbaum et al. 2014; Ghoshal and Gardiner 2021) (Fig. 6c).

Unfused *M.Sss*I has been expressed in tomato plants using the LhG4/pOP transactivation system, in which the inactive *pOP* promoter is trans-activated in the presence of the transcription factor LhG4 (Kumar et al. 2024). Thus, one of the plant parents carried the LhG4 under the control of the *pFIL* promoter while the other parent carried the methylase under the control of the *pOP* promoter. Members of the first progeny (F1) successfully expressed the methylase since they carried transgenes for both LhG4 and the methylase (Fig. 6d). The effect of *M.Sss*I on the genome methylation of the tomato plants was determined by sequencing the methylomes of the second (F2) and third (F3) progenies. In plants expressing the methylase, CG regions were hypermethylated compared to those in control plants which do not express the enzyme. The main target of *M.Sss*I were regions of euchromatin that were rich in genes which were mostly unmethylated in wild-type plants. Some genes displayed greater susceptibility to CG methylation near their transcription start site and this was associated with variable effects on gene expression. Tomato plants with novel genome methylation profiles were created by the heterologous expression of this bacterial methylase, and as the methylation was hereditable, this offers valuable potential for agricultural purposes (Kumar et al. 2024).

Plant growth-promoting bacteria (PGPB) are beneficial bacteria of great importance in plant breeding for sustainable agriculture. PGPB play important roles in plant growth and crop productivity, resistance to environmental stress and biocontrol. They can also secrete metabolites that induce changes in plant DNA methylation patterns (Doddavarapu et al. 2024). Inoculums of PGPB have been used to modify DNA methylation in plant root genes to promote plant growth (Fig. 6e). The PGBP *Bacillus* sp. and *Arthrobacte*r sp. have been inoculated into seedlings of *Phytolacca americana*, a plant that accumulates Mn and Cd and has bioremediation potential. Different methylation and expression patterns were found in the roots of inoculated plants compared to control plants and the PGPBs induced methylation in genes related to plant growth. Although the inoculums did not survive over the longer term, due to competition with the indigenous microbiome, the methylation patterns they induced persisted long-term after the inoculated bacteria had been eliminated, thus continuing to promote plant growth in the late phase of the plants’ development (Chen et al. 2022).

## Concluding remarks and future research

Bacterial DNA methylases are being developed as valuable tools in molecular and synthetic biology with applications across diverse fields including biotechnology and agriculture. These enzymes can be found as orphan solitary enzymes or as part of R-M systems, where they are associated with a corresponding restriction enzyme. The sequence specificity of bacterial methylases varies widely and can be used to target methylation with precision. Alternatively, methylation can be targeted to particular sequences by fusing a methylase domain to a molecule with the required DNA-binding specificity, such as a ZF, TALE or CRISPR/dCas9. Similarly, DNBPs can be fused to demethylases to provide sequence-specific demethylation. Targeted inhibition of enzyme activity can also be achieved using unfused DNBPs that bind to specific sequences and sterically block access of methylases or demethylases to those sequences, and the use of dCas9/gRNA to provide steric hindrance permits a site-selective blockade that has high specificity and is easily customized for different sequences by changing the guide RNA sequence (Sapozhnikov and Szyf 2021, 2023).

Bacterial methylases have been used to develop DNA assembly methods, improve bacterial transformation, and as tools in plant engineering. In DNA assembly, methylases can be used to methylate restriction enzyme sites and so prevent their digestion by restriction endonucleases activity during the assembly process. In this way, type II methylases have been used to ‘switch’ restriction enzyme recognition sites off (Lin and O’Callaghan 2018). The study of more methylases and their sequence-specificity and associated restriction enzymes may highlight further enzymes of particular value in DNA assembly (Flores-Fernández et al. 2024).

Bacterial transformation is an essential step for many processes in molecular and synthetic biology, genetic engineering and bacteriology. Pre-methylation of DNA can significantly improve its transformation efficiency by inhibiting digestion by host R-M system endonucleases. Further elucidation of host R-M systems, their enzymes and mechanisms will be valuable in the design of improved approaches for the transformation of bacterial strains that are currently difficult to transform (Ren et al. 2020). Improving bacterial transformation efficiency will allow the exploration of pathogenic mechanisms, virulence factors and antibiotic-resistance genes in clinical isolates (Ren et al. 2020; Ruivo et al. 2024). In bioindustry, improved transformation could expand the repertoire of useful host bacteria for metabolite production (Ren et al. 2020).

Methylation of plant DNA using fusion proteins formed between DNBPs and bacterial methylases has been investigated as a method for altering plant gene expression and phenotype. Bacterial inoculums with capacity to alter plant DNA methylation have also been investigated as tools for plant engineering. Methylation of plant DNA by bacterial methylases is hereditably maintained over plant generations, even in the absence of ongoing expression of the bacterial methylase. The use of bacterial methylases has the potential to control yield, disease and environmental resistance in agricultural crop plants (Mercé et al. 2020). Overall, bacterial DNA methylases are proving highly valuable in a wider range of applications and achieving a deeper and wider understanding of the repertoire of bacterial methylases is likely to be an important factor in the development of new methods and tools in molecular and synthetic biology across even more applications.

## Data Availability

All data generated or analyzed during this study are included in this published article.
